# Accreditation of the handheld 3-dimensional scanner and conventional photo images for area measurement

**DOI:** 10.1097/MD.0000000000035376

**Published:** 2024-02-09

**Authors:** Chao-Wei Chang, Meng-Chien Hsieh, I-Wen Lin, Rong-Fu Chen, Yur-Ren Kuo, Su-Shin Lee

**Affiliations:** a Division of Plastic Surgery, Department of Surgery, Kaohsiung Medical University Hospital, Kaohsiung, Taiwan; b Department of Nursing, Faculty of Medicine, College of Medicine, Kaohsiung Medical University, Kaohsiung, Taiwan; c Department of Surgery, Faculty of Medicine, College of Medicine, Kaohsiung Medical University, Kaohsiung, Taiwan.

**Keywords:** 3-dimensional, anthropometry, scanner, smartphone

## Abstract

Accurate assessment of wound areas is crucial in making therapeutic decisions, as the prognosis and changes in the size of the wound over time play a significant role. An ideal assessment method should possess qualities such as speed, affordability, accuracy, user-friendliness for both patients and healthcare professionals, and suitability for daily clinical practice. This study aims to introduce a handheld 3-dimensional (3D) scanner and evaluate its accuracy in measuring wound areas. Engineers from the Industrial Technology Research Institute in Taiwan developed a handheld 3D scanner with the intention of extending its application to the medical field. A project was conducted to validate the accuracy of this 3D scanner. We utilized a smartphone (Asus ZenFone 2 with a 13-million-pixel rear camera), a digital single-lens reflex digital camera (Nikon, D5000, Tokyo, Japan), and the 3D scanner to repeatedly measure square papers of known size that were affixed to the curved surface of life-size facial mask or medical teaching breast models. The “Image J” software was employed for 2-dimensional image measurements, while the “3D Edit” software was used to assess the “area of interest” on 3D objects. By using square papers with predetermined dimensions, the measurement-associated error rate (ER) could be calculated for each image. Three repeated measurements were performed using the “Image J” software for each square paper. The ERs of the 3D scan images were all below 3%, with an average ER of 1.64% in this study. The close-up mode of the smartphone exhibited the highest ER. It was observed that as the area increased, the ER also increased in the digital single-lens reflex camera group. The extension distortion effect caused by the wide-angle lens on the smartphone may increase the ER. However, the definition of a healthy skin edge may vary, and different algorithms for calculating the measurement area are employed in various 3D measurement software. Therefore, further validation of their accuracy for medical purposes is necessary. Effective communication with software engineers and discussions on meeting clinical requirements are crucial steps in enhancing the functionality of the 3D scanner.

## 1. Introduction

Accurate wound area measurements are crucial for assessing treatment effectiveness and implementing evidence-based medicine. Prognosis and changes in wound surface area over time often inform therapeutic decisions.^[1]^ Various methods have been used to record wound conditions and area, including manual drawing on paper, squared paper measurements, orthomorphic light projection, and the Visitrak system.^[2,3]^ With the advent of digital technology, digital cameras, including smartphones, have become widely used for photographing wounds, offering convenience in clinical practice for documenting and sharing images.^[4,5]^ Three-dimensional (3D) scanners have also been employed to survey human surface anthropometry.^[4,5]^ However, the use of 3D scanners has advantages and drawbacks.

The Visitrak system is a reported rapid and accurate technique for measuring wound areas in medicine, but its limitation lies in investigating small wounds or pressure ulcers of limited size.^[6]^ Digital cameras, including digital single-lens reflex (DSLR) cameras and smartphones, provide convenience for daily use, allowing easy discussion, documentation, and sharing among healthcare professionals.^[1]^ However, smartphone cameras with wide-angle lenses, designed to capture a larger visual field, may introduce peripheral distortions, particularly as the wound area increases. A comparison study in 2018 found that the Planimator app on the Android operating system exhibited better accuracy and precision than other devices and software.^[1]^ In recent years, 3D tools have become increasingly common for measuring wound areas, particularly for evaluating treatment effects on chronic wounds. However, the disadvantages of 3D scanning systems include high cost, potential missing data due to shading and movement artifacts, and the need for patient immobilization during scanning, posing a disadvantage compared to 2D photography.^[4,7]^

Accurate estimation of burn size is crucial in the treatment of major burn patients, but it remains challenging even for experienced burn care providers. Computerized models and objective methods have the potential to improve burn size estimations and enhance future burn care.^[8–13]^ Similarly, precise wound surface area measurements are essential for tracking the progression of chronic wounds and guiding therapeutic decision-making.^[14]^

To address the drawbacks of previous bulky 3D scanner systems, an ideal assessment method should be quick, affordable, accurate, patient-user-friendly, and suitable for daily clinical practice. Additionally, it should not require specialized training for operators.^[15]^ In this study, the authors describe a handheld 3D scanner and verify its accuracy.

## 2. Materials and methods

### 2.1. Introduction and collaboration between Industrial Technology Research Institute and Kaohsiung Medical University

A portable handheld 3D scanner was developed by engineers from the Industrial Technology Research Institute (ITRI) in Taiwan, with the aim of expanding its application in the medical field. To ensure the accuracy of this newly developed scanner, a collaborative project between ITRI and Kaohsiung Medical University was undertaken. Initially, the project focused on measuring various wound surface areas on patients. However, due to regulatory restrictions on medical equipment in Taiwan, direct measurements on patients were not permitted. Consequently, the 3D scanner had to undergo the medical equipment inspection registration process and obtain approval from the Institute Review Board before it could be used for human scanning. As a result, life-size face masks and breast teaching models were scanned as alternatives in this study.

### 2.2. Scanning process and software capabilities

The handheld 3D scanner utilizes a high-resolution digital video camera and infrared technology for distance detection. It is directly connected to a computer, which enables real-time visualization of 3-dimensional (3D) images on the monitor. The accompanying “3D Edit” software allows users to program and manipulate full-color 3D image objects by performing functions such as rotation, resizing, cutting, and deleting specific portions. The software also facilitates area and volume calculations based on user-defined regions of interest. The scanning process involves the central processing unit (CPU) of the computer performing calculations, identification, and fusion of each captured image. Therefore, a high-speed CPU and advanced graphics cards can enhance the scanning results.

### 2.3. Experimental setup and measurement techniques

For the purpose of validation, the authors employed a smartphone (Asus ZenFone 2 with a 13-megapixel posterior camera), a DSLR digital camera (Nikon, D5000, Tokyo, Japan), and the 3D scanner to measure the size-given square papers attached to curved surfaces of life-size facial masks and medical teaching breast models. Five sizes of colored square papers were used with areas of 2.25 cm^2^ (1.5 × 1.5 cm), 16 cm^2^ (4 × 4 cm), 25 cm^2^ (5 × 5 cm), 64 cm^2^ (8 × 8 cm), and 100 cm^2^ (10 × 10 cm). Smaller-sized papers (2.25, 16, and 25 cm^2^) were applied to the facial mask, while larger papers (64 and 100 cm^2^) were used on the breast model.

**Figure 1.**
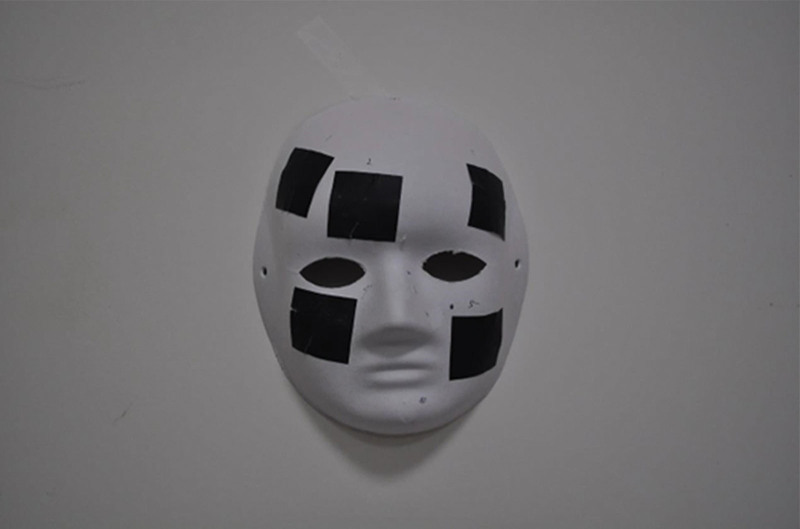
The life-size facial mask full-face view, with 4 × 4 cm square papers.

**Figure 2.**
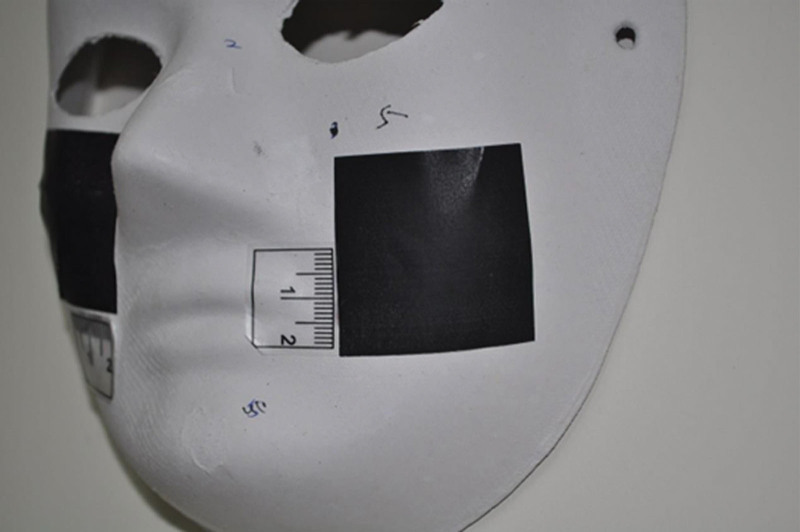
The left cheek 4 × 4 cm square paper photographed in the perpendicular direction.

**Figure 3.**
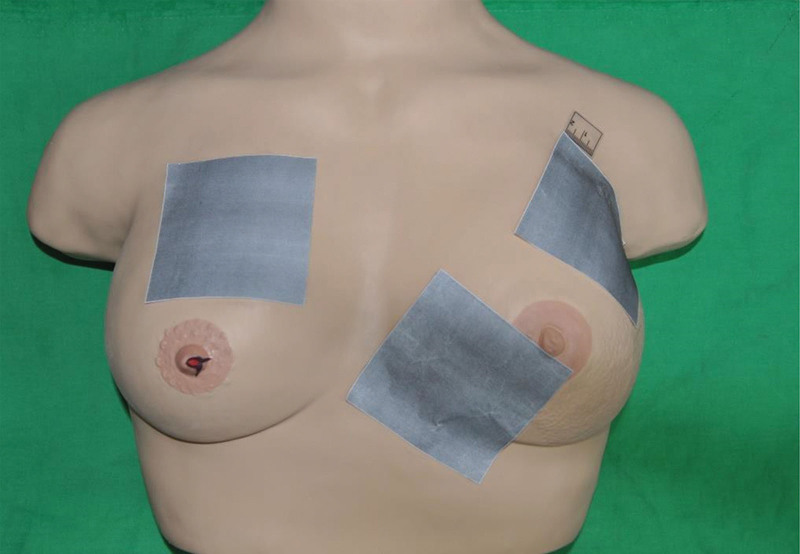
The breast teaching model with 8 × 8 cm square papers.

**Figure 4.**
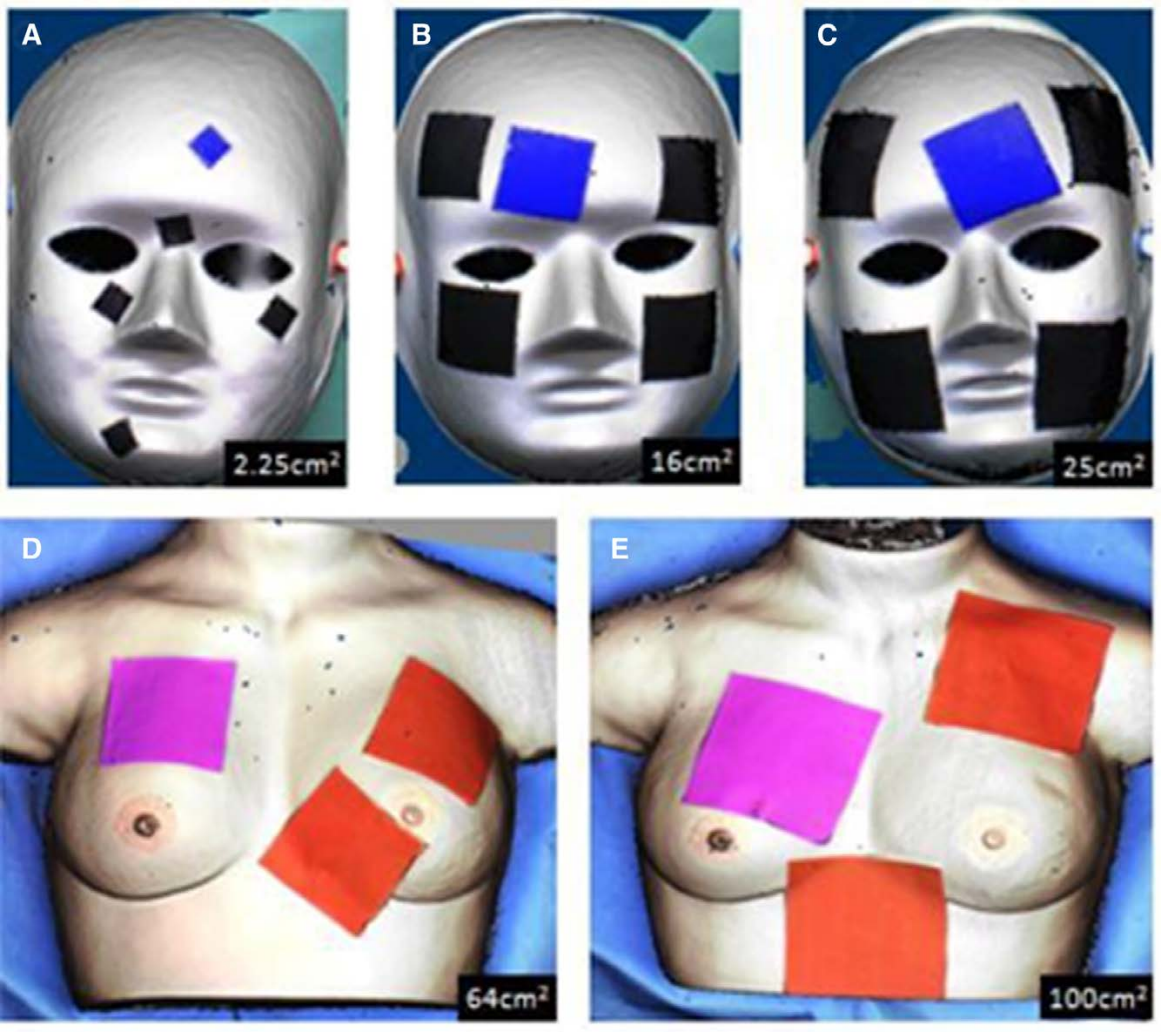
The 3D scan images of 5 different sizes of square papers on the facial mask (A, B, C) and on the breast model (D, E). (A) Area of 2.5 cm^2^. (B) Area of 16 cm^2^. (C) Area of 25 cm^2^. (D) Area of 64 cm^2^. (E) Area of 100 cm^2^. 3D = 3-dimensional.

The face mask was hooked to the wall (Fig. 1). The square papers along with ruler stickers were randomly applied on the curved surface of the mask. Each square piece of paper was photographed in the perpendicular direction (Fig. 2). The DSLR camera with a tripod was set at a close-up mode to allow clearly see the largest square paper view in each image. The smartphone captured pictures in the same direction as the DSLR camera. Additionally, the 3D scanner was employed to capture 3D images of the mask. The “Image J” software package, downloaded from the Internet (National Institutes of Health: Http://rsb.info.nih.gov/ij/download.html) was used to measure these square paper photographs. The areas of these square papers were measured by the research assistant (L.L. Hong). “3D Edit” software was used to measure the square paper area of these 3D images. For the assessment of larger areas, the breast model was used, which was placed on a horizontal table. Size-given square papers were pasted on the curved surface together with a ruler sticker (Fig. 3). Again, the smartphone, DSLR camera, and a 3D scanner were used to capture these images. For 2-dimensional (2D) photographs, the “Image J” Software was used to measure the area of interest. The 3D scan results were then uploaded into the “3D Edit” software system (Fig. 4). The user could select 1 specific color and the area of the same color gradient on the 3D object will be marked automatically as the “area of interest.” Then, we could measure the marked area directly.

### 2.4. Error rate calculation and comparison of 3D images and 2D photographs

The error rate (ER) for each image was calculated using the formula |*A* − *B*|/*B*, where *A* represents the measured area and *B* represents the true area. The average ERs of the 3D images and 2D photographs were determined and compared. The results are presented in Table 1 (supplemental digital content) demonstrating the accuracy of the 3D scanner in measuring wound surface areas. By comparing the ERs between the 2 modalities, it is possible to evaluate the performance of the handheld 3D scanner in relation to traditional 2D photography. The findings from this analysis contribute to the understanding of the scanner’s accuracy and its potential benefits for wound assessment and management.

**Table 1:** The measurement ER in different type of camera.

Area	3D scanner			2D photography (ZenFone2)			2D photography (Nikon)		
	ER (%)	M1	SD	ER (%)	M2	SD	ER (%)	M3	SD
	3.11			3.11			10.2		
							2		
	0.89			24			20.0		
2.25	2.67	2.756	1.673	18.22	16.89	8.01	14.22	10.93	6.37
	1.78			19.56			4.44		
	5.33			19.56			5.78		
	2.06			8.31			5.44		
	1.81			10.38			9.50		
16	2.75	1.396	1.1622	7.19	6.76	2.81	3.69	5.2	2.98
	0.18			3.50			1.44		
	0.18			4.44			5.94		
	1.44			10.12			4.20		
	0.92			3.44			5.88		
25	2.4	1.872	1.1542	0.40	11.79	14.36	6.16	7.68	7.50
	0.96			8.48			1.48		
	3.64			36.52			20.6		
							8		
	0.03			7.81			9.28		
	1.67			6.56			11.7		
64		0.643	0.8947		6.61	1.18	2	10.5	1.22
	0.23			5.45			10.5		
							0		
	2.27			2.35			14.0		
100		1.54	0.6393		3.94	2.42	1	10.34	3.23
	1.08			6.72			9.11		
	1.27			2.75			7.91		
Average error rate	1.64%			9.20%			8.93%		

Supplement data: 3D scanner raw data, http://links.lww.com/MD/L298.

ER = error rate, M1 = mean of error rate in 3D photography, M2 = mean of error rate in 2D photography (ZenFone2), M3 = mean of error rate in 2D photography (Nikon D5000), SD = standard deviation.

## 3. Results

**Figure 5.**
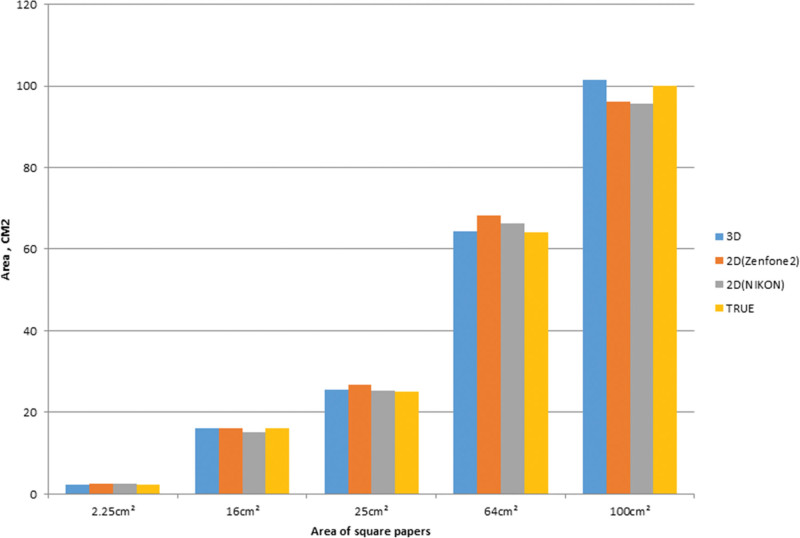
The average measured area in different groups with 2D camera and 3D scanner. The yellow bar represents the true area of the papers. The blue, orange, and gray bars represent the area measured taken by the 3D scanner, smartphone camera, DSLR camera, respectively. 2D = 2-dimensional, 3D = 3-dimensional, DSRL = digital single lens reflex camera.

**Figure 6.**
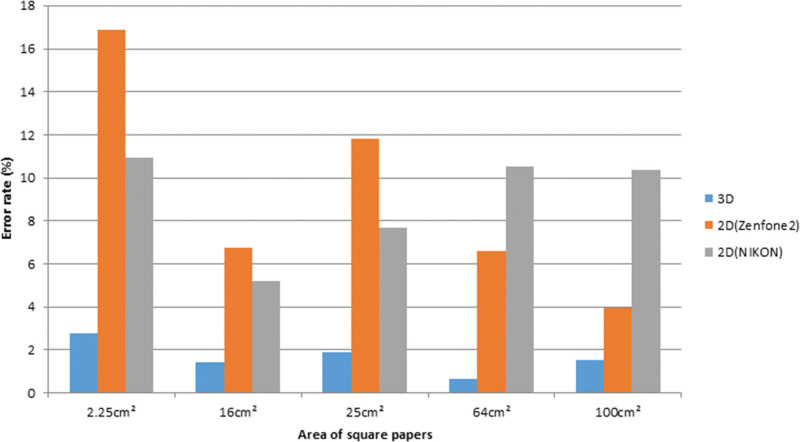
The averaged error rate measured by 2D camera and 3D scanner in different size groups. The error rate of the 3D scanner group is always lower than the error rate of the 2D groups. For the 2.25 cm^2^ area, the 2D (ZenFone2) group has the highest error rate than the other 2 groups. 2D = 2-dimensional, 3D = 3-dimensional.

In the facial mask groups with areas of 2.25, 16, and 25 cm^2^, 5 square papers were utilized for each group. Each square paper underwent 3 repeated measurements using the “Image J” software. Similarly, in the breast model groups with areas of 64 and 100 cm^2^, 3 square papers were employed, and 3 repeated measurements were conducted. The obtained results of the measured areas from both the 2D and 3D images were compared and presented in Figure 5. Since size-given square papers were deliberately used, the ERs of the 2D and 3D images could be calculated for each case. The average ERs for different size groups are presented in Table 1. Overall, the 3D scanner exhibited the lowest ER when compared to the smartphone and DSLR cameras. In this study, all ERs of the 3D scan images were below 3%. Notably, the smartphone’s close-up mode exhibited the highest ER. Additionally, it was observed that as the area increased, the ER also increased in the DSLR camera group. Figure 6 provides a visual representation of the average ERs derived from images captured using different types of cameras.

## 4. Discussion

Nowadays, the use of smartphones for capturing clinical images has become convenient in daily clinical practice. These images can be immediately shared with other doctors for discussion or included in medical records. In comparison, DSLR cameras require a connection to a computer for image transfer. However, both smartphone and DSLR cameras offer portability, affordability, and ease of operation. Nevertheless, when using 2D photography to measure wound size, the challenge arises from treating a curved surface as flat. Additionally, this study identified that photographing small targets using the close-up mode resulted in higher inaccuracies. Specifically, in the 2.25 cm^2^ group, the smartphone camera and DSLR camera exhibited ERs of 16.89% and 10.93%, respectively. Our measurements (Table 1) revealed an inherent average ER of 8.9% to 9.2% for 2D photogrammetry. Generally, 3D scanners are not commonly used in the medical field. Existing whole-body 3D scanners are bulky and complex, limiting their practical clinical application. In the past, markers were necessary to facilitate landmarking and data collection.^[5]^ However, in recent years, handheld 3D scanner systems have been developed and made commercially available. One major advantage of the 3D method is its ability to determine wound perimeter, area, and volume.^[15]^ However, since different 3D measurement software employs various algorithms for calculating area/volume, further validation of their accuracy for medical purposes is required.

Pavlovcic and Jezersek^[16]^ presented a measuring system that utilizes a commercial DSLR camera and a light pattern projection system. This system operates based on triangulation and structured illumination principles, enabling handheld measurements. The authors claimed an accuracy of over 95% for area measurement and 93% for volume measurement. However, our study found that the 3D system employed herein achieved an average ER of 1.64% and outperformed Pavlovcic system in area measurement.

Computed tomography or magnetic resonance imaging (MRI) have been previously employed for objective volume measurement.^[17]^ However, computed tomography radiation exposure and the financial cost of magnetic resonance imaging limit their suitability for repeated examinations. Therefore, the 3D scanner system emerges as a promising tool for future clinical use.

Both the 3D scanner and 2D camera have demonstrated their effectiveness as noninvasive tools for capturing high-quality images of human body parts without causing discomfort or requiring direct physical contact with patients. In this study, scanning the breast model took an average of 2 minutes using the 3D scanner, while the 2D camera required only 1 second. However, for calibration purposes, the 2D method often requires placing a ruler next to the object as a reference. Moreover, 2D photography necessitates additional analysis using software such as “Image J” to approximate the size of the area of interest. Our results support the higher accuracy of the ITRI 3D scanner system compared to the 2D method. Furthermore, the 3D scanner enables volume measurements, which the 2D camera cannot perform. Future research will focus on evaluating and studying the accuracy of volume measurement using the ITRI 3D scanner.

Despite the numerous advantages, there are some drawbacks associated with the 3D scanner system. Firstly, the handheld scanner requires connection to a computer via a transmission line and power cable, which limits its portability during scanning. Secondly, any movement of the object during scanning compromises the image quality. To mitigate these issues, the current solution involves utilizing a high-speed CPU and advanced graphics card to reduce the scanning duration.

The ITRI handheld 3D scanner has recently received approval from the Institute Review Board committee to conduct a human trial. The authors of this study are currently conducting additional tests to ensure the quality control of the handheld scanner. Effective communication with software engineers and discussions regarding meeting clinical requirements are crucial for enhancing the functionality of the 3D scanner. In addition to measuring wound area, the 3D scanner can also record changes in volume, making it useful for documenting preoperative and postoperative volume/area in various surgical procedures such as blepharoplasty, rhinoplasty, liposuction, fat grafting, and breast augmentation. The potential benefits of the 3D scanner for broader medical applications are limitless. With faster CPUs and state-of-the-art graphics cards, this handheld 3D scanning system can provide valuable references to clinicians and enhance the quality of healthcare in the future.

3D scanner raw data, Supplemental Digital Content, http://links.lww.com/MD/L298.

## Author contributions

**Data curation:** Chao Wei Chang.

**Methodology:** Chao Wei Chang.

**Project administration:** Chao Wei Chang.

**Software:** Chao Wei Chang.

**Writing—original draft:** Chao Wei Chang, Meng-Chien Hsieh.

**Conceptualization:** Meng-Chien Hsieh, I-Wen Lin, Yur-Ren Kuo, Su-Shin Lee.

**Validation:** Meng-Chien Hsieh, Rong-Fu Chen, Su-Shin Lee.

**Writing—review & editing:** Meng-Chien Hsieh, Rong-Fu Chen, Su-Shin Lee.

**Formal analysis:** Su-Shin Lee.

**Investigation:** Su-Shin Lee.

**Supervision:** Su-Shin Lee.

**Visualization:** Su-Shin Lee.

## Supplementary Material


